# The legacy of a surgeon - in memoriam Dr. İsmail Demir

**DOI:** 10.1007/s00423-024-03467-w

**Published:** 2024-09-17

**Authors:** Ihsan Ekin Demir, Elke Demir, Mert Erkan, Güralp O. Ceyhan, Helmut Friess

**Affiliations:** 1 Department of Surgery, Klinikum rechts der Isar, School of Medicine, Technical University of Munich, Munich, Germany; 2 Department of General Surgery, HPB-Unit, School of Medicine, Acibadem Mehmet Ali Aydinlar University, Istanbul, Turkey; 3 Else Kröner Clinician Scientist Professor for Translational Pancreatic Surgery, Munich, Germany

**Keywords:** Principles, Surgery, Patient care, Cure, Lesson, Turkey, Cardiothoracic, Vascular, Esophagectomy

## Abstract

In this perspective article, we highlighted some special aspects of being a surgeon that are typically not taught in medical training. Departing from a real and personal story, the present manuscript is intended to communicate how surgery imbues us doctors with an unparalleled degree of satisfaction, gratification, meaning and fulfilment, like no other field of medicine.

## Main text

“Please allow me to kiss your hand…”

In my native Turkey, a hand kiss is given not primarily as a sign of courtesy and admiration to women, but rather as a high sign of respect to elderly or teachers.

I was overwhelmed that a man in his 70s approached me, back then in 2005 a 22-year old medical student, said the entry sentence of this text and attempted to kiss my hand.

**Fig. 1:**
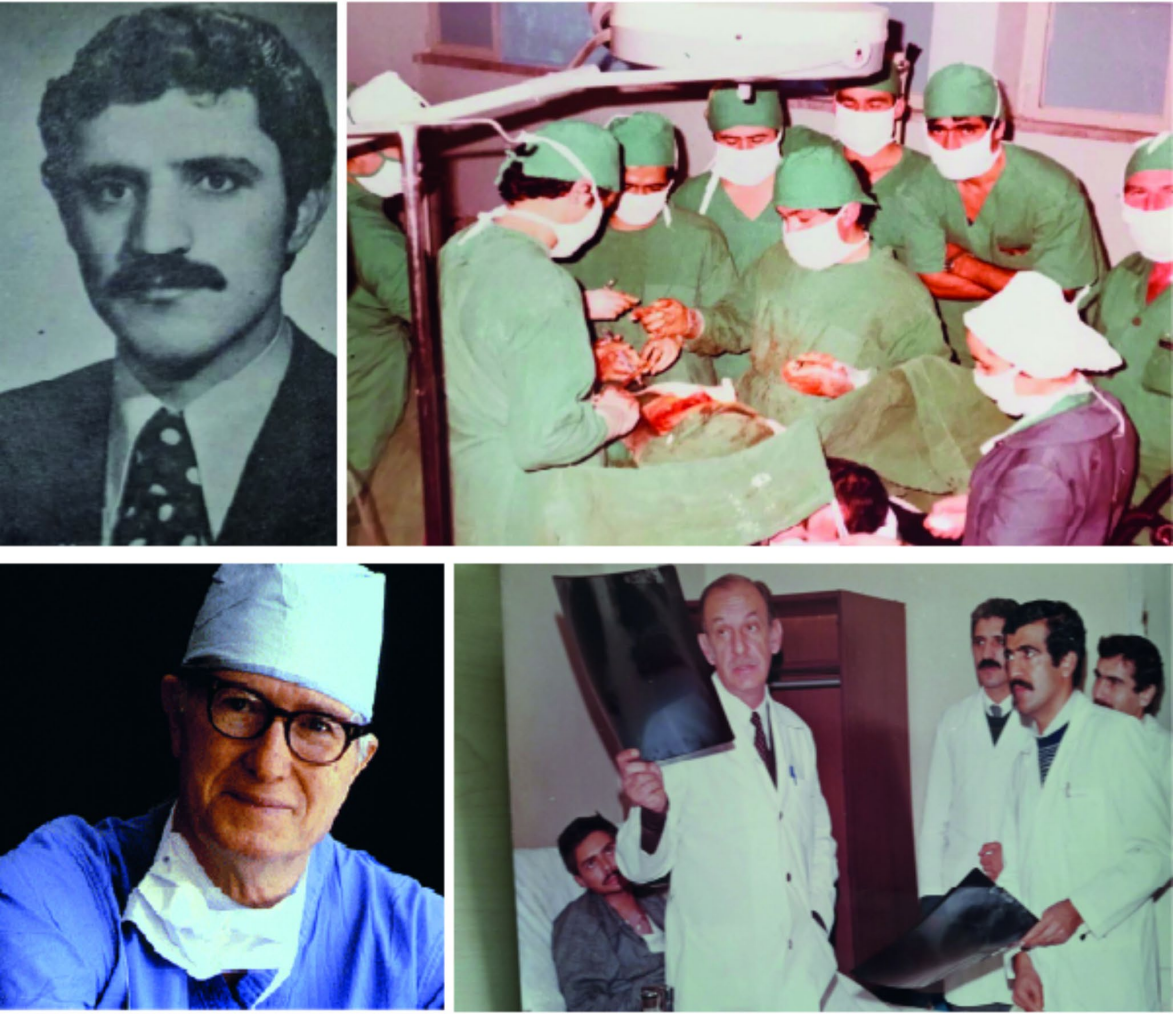
**A**. Dr. Ismail Demir in his medical school years. **B**. During a thoracotomy for a lung cancer (left on the image), **C**. Dr. James Hardy, who performed world’s first human lung transplantation in 1963. **D**. Prof. Isin and Dr. Demir, and the thoracic surgical team analysing a chest X-ray during a surgical round

In the summer of 2005, when I had finished the 6th semester of medical school in Heidelberg and was considering to apply for an exchange programme between Heidelberg and the UMass Medical School, I decided to more closely get in contact with the medicine in my native Turkey. I am deliberately writing “more closely”, because I grew up listening to the way medicine and especially surgery is performed in high-volume state hospitals in Turkey (Fig. 1). My father, who was trained as a thoracic and cardiovascular (TCV) surgeon in Antalya by Dr. Erol Isin, a fellow of Dr. James D. Hardy in Jackson, Mississippi [1], (Fig. 1), moved in mid 1980s as part of his obligatory state service to the state hospital of Kocaeli, the neighbouring city of Istanbul, as the only TCV surgeon of the then half-a-million city. In Turkey, such state hospitals (still) harbour extremely busy surgical services and emergency ORs due to a relatively high burden of traffic accidents, stabbing or shooting gun wounds. I feel that, if a surgeon has not ever participated in such a high case-load trauma practice, s/he definitely missed something, and the colleagues who bring about medical wonders in these services have my deep respect and admiration.

Naturally, my father was immersed in clinical work, operating nearly every day on highly complex emergency and elective patients, who would – today – require highest level ICU care, but had, in the existing conditions, to be immediately taken care of by him, the only TCV surgeon of the city. And for sure, he missed to spend sufficient free time with me and my mother. Yet it seems to me that, he still managed to build extremely strong ties with his family although he was working nearly all the time.

One key reason for this successful bonding was the fascination for his profession that he transmitted to me and my mother every day. I grew up listening to surgical terminology ever since I can remember: I learned the names of many surgical instruments from him and his surgical atlases during elementary school, got acquainted as a 10-year old with the names of surgical procedures like aorto-bifemoral bypass, Thompson procedure for lymphedema, or Ivor-Lewis esophagectomy thanks to him as he told me about these procedures in the evening when he made it to dinner at home.

Thinking back, it was, however, not these fancy technical terms or objects that captivated me, but other aspects of surgery he emphasized, some that are even today, after nearly 30 years, not sufficiently articulated in our profession.

How could a surgeon perform the most complex types of procedures of his discipline in a state hospital with limited resources and still with low morbidity? Certainly, a considerable amount of experience, careful case selection, and team work, take a big role in this success. Such success stories are not unique and encountered every day in many parts of this planet, including the low-income countries.

But there is more to it: he agreed that the above mentioned aspects were not the most deciding ones to surgical success.

*A patient is cured, only if you care and care and care*.

In the highly modernized hospitals and wards we work, as part of the large doctors’ and nurses’ teams, and in the era of increasing standardization and technical perfection, how much time do we actively spend to take care of our patients? How much of a work-life balance are we, surgeons, still ready to sacrifice for a high-quality, personal care toward the surgical patient?

*Undoubtedly, in surgery, the patients you lose will teach you the biggest lessons*.

The life of a surgeon takes a different course once s/he finishes residency and gets the primary responsible surgeon for the patients. This means that, s/he decides on the indication for surgery, performs it, and takes full responsibility of the postoperative course. In contrast with what young surgeons might believe, the “loss” of a patient occurs not always during or after surgery, but often *before* surgery, when the decision to perform surgery is taken at the wrong time, or in an insufficiently understood or worked-up patient. Such bitter cases, regardless of our level experience, still tend to recur in the progress of our career, albeit at decreasing frequency.

*Who else should perform it, if not you?*

We surgeons do often ask ourselves “should I do it or not?” during an operation, or before or after it. An extreme example is a surgeon on call who is called to the ER to treat a patient with a stab wound in the heart. This is, in many European countries, not a frequently seen case. Imagine being the resident or young fellow (or even young attending) who is in charge of this patient, and imagine you have, due to the nature of this severe injury, only seconds to minutes to act. It is likely that you have never performed an emergency thoracotomy, e.g. a clamshell thoracotomy, in the real-life setting, and there is no training that can make you a priori confident enough to perform such an urgent thoracotomy with ease in such a situation. If the indication for it is given, and if you have acquired sufficient basic surgical skills, it is likely that you yourself will be the only obstacle in front of performing an emergency thoracotomy in such a hyperacute situation. Hesitance can in such cases lead to loss of time, and to loss of the patient; therefore, who else should do it, if not you?

*Surgeons live in the vicinity of God*.

There is indeed a boundary between life and death. Often, during complex surgery, or in unexpected complicated surgical moments, surgeons tend to feel this boundary on their skin, in their mind and in their heart, possibly even more than the patient does. While walking on this boundary, surgeons may in themselves turn to a higher power and ask there for help and strength. At the very border between life and death, this is a natural human reaction. To tackle such a difficulty, the team must operate in harmony and fully trust each other. In most cases, the team succeeds, and sometimes, it cannot save the patient. This is the natural attribute of this profession. Yet this feeling of mutual confidence and harmony, both at the service of a divine goal, i.e. to save a life, imbues the team with a sense of fulfilment. And this is truly unique for surgery.

*Surgery is actually not a profession, but a lifestyle*.

Coming back to the 70 year old man, he told me that my father had operated on him due to esophageal cancer during 1990s and that he is still alive thanks to him. His gratitude motivated him to show his unique way of respect now to the (future doctor) son of his doctor. Interestingly, during those weeks I spent in the my father’s hospital in 2005, many of my father’s colleagues embraced me at the very first encounter. Although Turkish people are warm by their nature, this was more than that, making me feel their way of expressing love and respect to a unique surgeon.

I then went to my father’s room in the hospital to tell him about my emotional encounters of the day. He had stopped performing surgery eight years ago due to a ruptured cerebral aneurysm that left him with a left-sided paralysis, but still continued to perform outpatient clinic and administrative duties. My father was certainly a very emotional person, who cannot easily find any words to say in emotional situations. When I told him about my utmost emotional encounters of the day, he remained rather calm and replied “*if you love surgery*,* it is actually not a job*,* but a lifestyle.”*

I sometimes ask myself: but what happens to us surgeons, if we stop operating, e.g. upon retirement, due to health issues or other life circumstances? Do we then lose our lifestyle? Is everything then over?

At first glance, may be…but then I remember my encounters from the summer of 2005: the dazzled medical student, feeling the love of people I had never met before, perceiving the border of life and death, and the people you can inspire….

At the end, it seems to me that it is all worth for this legacy.

## Data Availability

No datasets were generated or analysed during the current study.
